# Trajectory analysis of *Mythimna Loreyi* migration into Korea using the HYSPLIT model

**DOI:** 10.1038/s41598-025-07876-9

**Published:** 2025-07-01

**Authors:** Juhyeong Han, Sunghoon Baek, Xue-Jing Wang, Chun-Sen Ma, Kwang-Hyung Kim

**Affiliations:** 1 Interdisciplinary Program in Agricultural and Forest Meteorology, Seoul National University, Seoul, 08826 Korea; 2 Department of Industrial Entomology, National College of Agriculture and Fisheries, Jeonju, Korea; 3 School of Life Science, Institutes of Life Science and Green Development, Hebei University, Baoding, 071002 People’s Republic of China; 4 Department of Agricultural Biotechnology, Seoul National University, Seoul, 08826 Korea; 5 Research Institute of Agriculture and Life Sciences, Seoul National University, Seoul, 08826 Korea

**Keywords:** Migratory moths, Moth trajectory, HYSPLIT, Loreyi leafworm, *Mythimna Loreyi*, Plant ecology, Atmospheric science, Entomology

## Abstract

Migratory moth species have a negative impact on agriculture in Korea, causing economic crop losses for farming households. The moths fly a considerable distance from their origins and settle in Korea, where they live for several generational cycles, feeding and laying eggs until the arrival of cold winter hinders their overwintering. Therefore, it is important to determine the timing and location of the moths’ departure and arrival to prevent potential harm from invasive moths. The goal of this study is to select the most likely origin and destination of the migratory moths by using the Hybrid Single-Particle Lagrangian Integrated Trajectory (HYSPLIT) model when there is limited moth occurrence data at the potential origin and destination. The HYSPLIT model was adopted and modified to investigate the potential trajectory paths of the loreyi leafworm (*Mythimna loreyi*), using atmospheric data. Occurrence data of *M. loreyi* was collected from 18 trap sites in Korea and five in China, and fed into the HYSPLIT model to conduct backward and forward trajectory analyses. As a result, we successfully prioritized the most likely origins and destinations of *M. loreyi* in China and Korea, respectively, which has potential implications for subsequent validation of moth migration, using genotypic and phenotypic characterizations. In conclusion, our results show that it is feasible to use the HYSPLIT model simulation for the initial screening of the most likely origins of migratory moths, leading to more efficient and effective validation study of insect migration between countries.

**Supplementary Information:**

The online version contains supplementary material available at 10.1038/s41598-025-07876-9.

## Introduction

Migratory moth species travel long distances. They encroach and outbreak in other countries causing serious impacts on domestic agricultural products that are economically important. Numerous instances of non-native species have been documented in Korea. It is reported that *Spodoptera frugiperda* was found in Northeastern Jeju in 2019, causing damages to at least four corn fields^1^. *Lycorma delicatul*, which were originally found in South China, India, and Vietnam and known to damage grapes, peaches, and pears, have also been found in Cheongju, Wanju, and Chuncheon^2^.

As it is impossible to block the inflow of these types of migratory moths, there needs to be proactive research on their migration, especially predicting the timing and location of arrival, to facilitate the immediate responses of controlling the moths. Due to their limited flight capability, these moths make use of strong wind flow in the upper atmosphere to be able to fly long-distances. Therefore, atmospheric trajectory analysis can be beneficial to simulate the moths’ trajectories.

Our focus in this research is on the loreyi leafworm *Mythimna loreyi*, a Noctuidae (nocturnal moth species), that has been continuously witnessed in Korea. *M. lorey* was reported to have been found first in Korea in 1982^3^. Since then, *M. lorey* larvae have been reported to cause damage to maize fields in Hadong between 2019 and 2020^4^. *M. loreyi* was reported to have been captured in Suwon^5^ and also observed in Gochang^6^. Although no significant crop damage caused by *M. loreyi* has been reported so far, it is crucial to understand the species’s migratory patterns and timing for implementing timely proactive measures to prevent significant damage in the future, such as crop yield losses, reduced crop quality, the spread of infestations, and ecological disruption.

The dynamics of insect herbivores are being and will be affected by global warming, both in terms of temporal and spatial changes^7,8^. This gives uncertainty to the study of migratory moth pathways and intensity. Warmer temperatures can lead to an increase in moth populations in their native or original habitats^9^. Also, the warming of winter temperatures in the arrival locations may result in the overwintering of the migratory insect species, as they can now survive upon their arrival due to more hospitable conditions^10^.

The Hybrid Single-Particle Lagrangian Integrated Trajectory (HYSPLIT) model was developed by the Air Resources Laboratory (ARL), National Oceanic and Atmospheric Administration (NOAA), USA to simulate how substances move through the atmosphere from a local to global scale^11^. As HYSPLIT can compute both forward and backward trajectory simulations from a starting and end point, respectively, there have been various application studies using this model, such as migratory moth trajectory or dispersion analysis. Forward trajectory refers to the chronological forward movement of air flow (e.i., from the past to the present), on the other hand, backward trajectory indicates the opposite, anti-chronological movement of the air parcel (e.i., from the present to the past). Seasonal migration of *Spodoptera frugiperda* first emerged in Texas and Florida, that potentially jeopardizes maize industry in Alabama-Georgia and Pennsylvania^12^. Also, Weather Research and Forecasting (WRF) model was used to improve accuracy of trajectory simulation for the migration of *Helicoverpa zea* in Texas, USA^13^. The ARL website was visited for the HYSPLIT model simulation through the Real-time Environmental Applications and Display System (READY) to research the re-invasion of *Bactrocera dorsalis* in Japan^14^.

Migratory moths can travel between regions by following prevailing wind patterns associated with global atmospheric circulation, primarily the westerlies at the latitude where Korea is situated. Thus, we hypothesized that, using backward trajectory from the locations in Korea where the moths are captured, it would be possible to pinpoint the most plausible origins of the moths with the HYSPLIT. Once we simulate where the moths come from, we could collect moth sampling data from the identified origins for further validation. On the contrary, conducting the HYSPLIT forward trajectory analyses using the collected data from the origin would inform where the moths might fly across the Korean Peninsula. Subsequently, by comparing moth occurrence data in Korea and the forward trajectory results from China, we tried to narrow down trap locations where *hit* (true positive cases where the modelled trajectory passed the trap location and the moth species was actually captured in the trap) occurred in both countries during corresponding time periods, which later become the candidate locations for the subsequent targeted validation of moth migration by other researchers.

The focus of this study, simulating the flying pathways of *M. loreyi* by using the HYSPLIT model, is slightly different from other studies mentioned above – we attempted to generate a GIS object containing regional information that can be used to further validate the moth migration using various genotypic and/or phenotypic methods in the subsequent studies. Therefore, the objective of this research is to understand the potential trajectory paths of the migratory *M. loreyi* using the HYSPLIT model, and thus to show a feasibility of providing an economic and efficient way of subsequent validation of the moth migration.

## Data and methods

### Data collection

Moth occurrence data is needed as the initial point for running the model trajectories. For the HYSPLIT model simulation, moth occurrence data for April, May, and June was used as an input data for forward and backward trajectory simulations from China and Korea, respectively.

#### Moth occurrence data in Korea

**Fig. 1:**
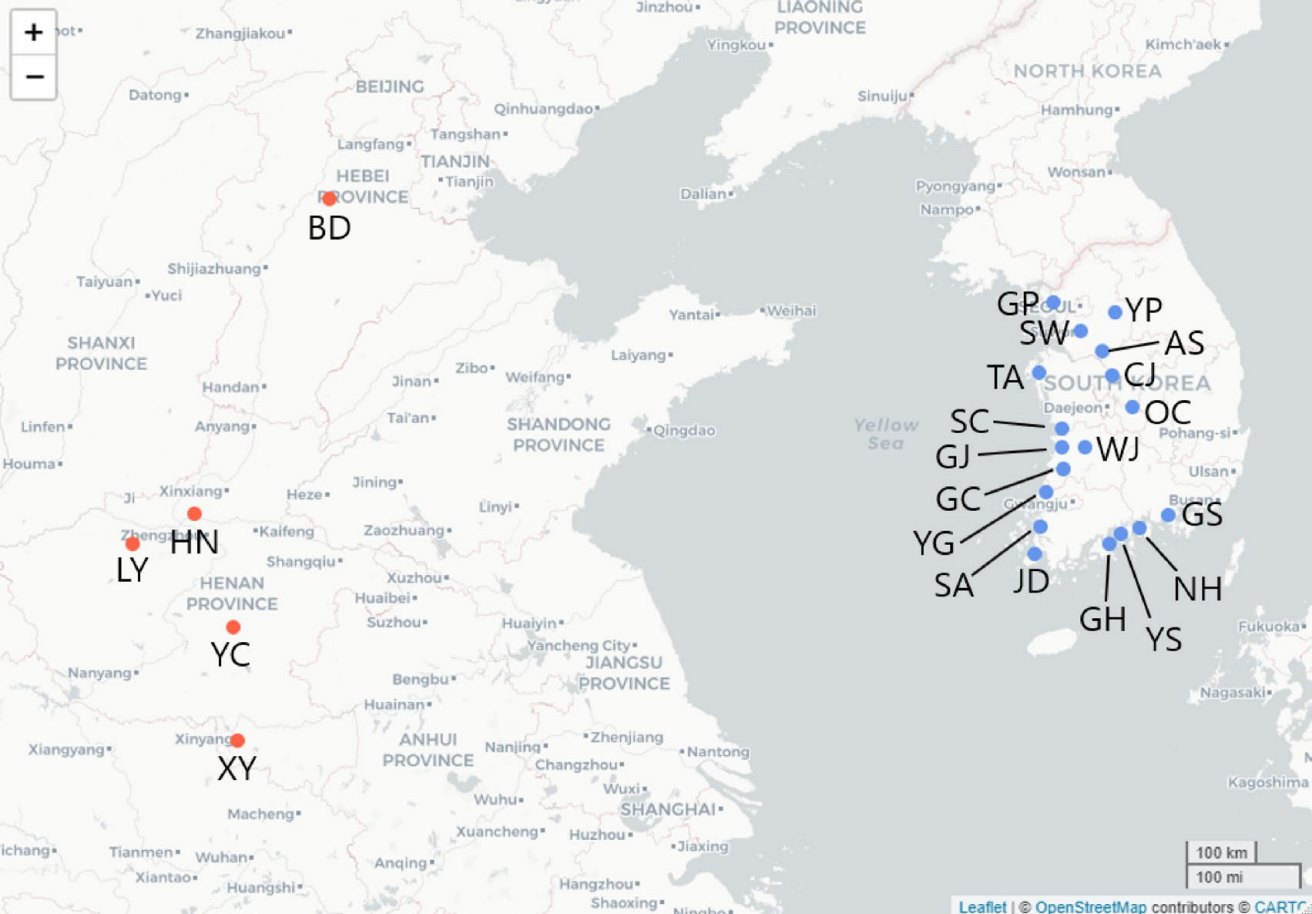
Trap locations marked on a map. Blue dots indicate 18 locations in Korea including Gimpo (GP), Yangpyeong (YP), Anseong (AS), and Suwon (SW) in Gyeonggi-do; Taean (TA) and Seocheon (SC) in Chungcheongnam-do; Cheongju (CJ) and Okcheon (OC) in Chungcheongbuk-do; Gimje (GJ), Wanju (WJ), and Gochang (GC) in Jeollabuk-do; Yeonggwang (YG), Shinan (SA), Jindo (JD), Goheung (GH), and Yeosu (YS) in Jeollanam-do; Namhae (NH) and Goseong (GS) in Gyeongsangnam-do. Red dots show five locations in China including Luoyang Academy of Agricultural and Forestry Sciences (LY), Baoding Station (BD), Xinyang Railway Station (XY), Yancheng Railway Station (YC), and Modern agricultural science and technology experimental demonstration base of Henan Academy of Agricultural Sciences (HN).

The data was collected in Korea from April to November in 2022, during which the moths were the most active in the agricultural fields. We used funnel traps for the sampling of the target moth, *M. loreyi*. The traps include a small compartment where a lure with pheromones is installed to attract male moths. Two sets of traps were installed in each trap site, and they were about 5 m away from each other. The traps were about 110 cm tall after installation. This data was collected from 18 trap locations in Korea (blue dots in Fig. 1), and was collected approximately once a week, starting from April 3. Due to the restricted time and resources, it was impossible to visit all the sites on the same day, thus the moth samples were gathered with a few days gap. As we did not check the trap everyday, we had to assume that the moths were captured sometime between two trap inspections.

We hypothesized that the moths migrate from China to Korea during the moth sampling period, between April and June. If the moths arrive in spring and reproduce several generations in Korea, we consider that the moths that are captured within Korean territory after June may either be newly migrated or the offspring of the initial spring migrant. Therefore, it is difficult to distinguish between the two groups of populations. We did not conduct the trajectory analysis where no moths were collected in certain regions on specific dates.

#### Moth occurrence data in China

Due to the westerlies, the most likely origin of the target moths is China. Thus, the moth occurrence data was collected from five locations in China (red dots in Fig. 1). We chose these areas in the eastern part of China, because they are known habitats of the target moths. The temporal resolution of this dataset is daily. We used this data as starting points to run forward trajectories towards the Korean Peninsula and see if the result matches our data collected in Korea. We used April, May, and June data due to the same reason as mentioned above.

### b. Model simulation

#### Atmospheric data

**Fig. 2:**
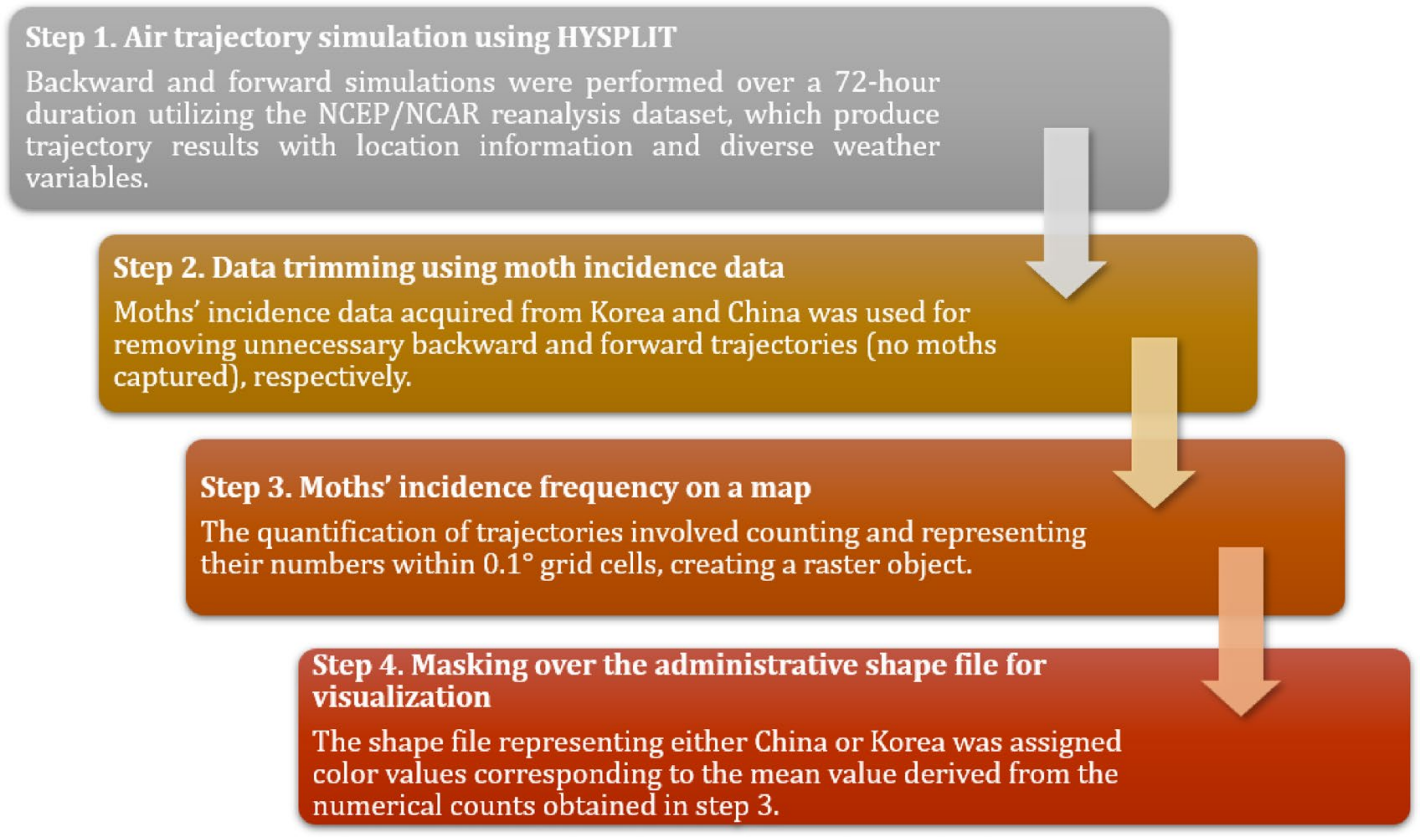
Modeling process explained in four steps. This process was applied for both backward and forward trajectory analyses in the study.

Weather input data is required to get trajectories using the HYSPLIT model. The ARL provides different sorts of data on the website. The data can be downloaded at https://www.ready.noaa.gov/archives.php. Among the available data, we used the NCEP/NCAR reanalysis data for the HYSPLIT modeling - the first process described in Fig. 2. The NCEP/NCAR reanalysis data contain variables – pressure, temperature at 2 m, U- and V-component of wind at 10 m, and total precipitation (6-h), geopotential height on the surface level, and upper atmospheric temperature, pressure vertical velocity, and relative humidity on the upper level. By obtaining spatial and temporal information of an air parcel from the reanalysis data, we pinpointed the location and height of the air parcel at a specific time, thus acquired trajectory information using the HYSPLIT model.

#### Modeling process

The first step of the HYSPLIT modeling (Step 1 of Fig. 2) is to run the HYSPLIT model to simulate backward and forward trajectories from the sampling sites in Korea and China, respectively, during a certain period at a certain location. The model can be run online in the NOAA ARL website or PC Windows-based version. However, because it required multiple repetition of tasks manually to perform multi-spatiotemporal analysis, we employed the HYSPLIT model using R software (version 4.2.2)^15^ and modified the script to enable air trajectory simulations from multiple locations and time. We used *splitr* package to run the model using R. It helps download meteorological data and analyze both trajectory and dispersion of substances, such as particulate pollutants or insects, using model simulations in R. To be able to run for multiple locations, we added an iteration for the *hysplit_trajectory* function of the *splitr* package. By running HYSPLIT model in R using the NCEP/NCAR reanalysis data, we can obtain simulation result data as well as variables like potential temperature, ambient air temperature, rate of rainfall, mixing depth, relative humidity, specific humidity, mixed layer depth, terrain height, and downward solar radiation flux.

A backward air trajectory was simulated once every week using the NCEP/NCAR Reanalysis data with daily temporal resolution and 2.5 × 2.5 spatial resolution. Model simulation duration was 72 h considering the physiology of the moths^16^. The atmospheric height at the starting point is 1,000 m above ground level (AGL)^17^, where jet streams blow, and the maximum model height was set to 20,000 m. The daily hours range from 0 to 23, meaning the simulations are conducted in the hourly scale. Each trajectory simulation consists of 72 hourly runs over a 72-hour period on the days when moth samples are collected. After this first step, we obtained the simulation result as a data frame, which was used in the next steps. The HYSPLIT parameters used in the study are delineated in Table 1. This first step consists of around 30 user-defined functions, and the details of them are described in splitr GitHub for public uses (https://github.com/rich-iannone/splitr).

**Table 1:** Model parameter description and settings used for the *hysplit_trajectory* function in the HYSPLIT model simulations.

Model Parameters	Description	Setting	Unit
lat, lon	coordinate information (latitude and longitude)	in decimal degree^1)^	°
height	the height at the start of model simulation	1000	m AGL
duration	model simulation duration	72	hours
days	model simulation days (1 or more consecutive)	“YYYY-MM-DD”	-
daily_hours	a vector of daily hours for initiations of runs in (a) given day(s)	0 to 23	hour
vert_motion	vertical motion^2)^. 0 indicates “input model data”	0	-
model_height	maximum model height (boundary)	20,000	m
direction	forward or backward trajectory simulation	“forward”/“backward”	-
met_type	data type - reanalysis, GDAS^3)^0.5, GDAS1, or GFS^4)^0.25, etc.	“reanalysis”	-
^1)^ location in formation is set as a separate dataframe			
^2)^ vert_motion: 0 - input model data, 1 - isobaric, 2 - isentropic, 3 - constant density, 4 - isosigma, 5 - from divergence, 6 - remap MSL to AGL, 7 - average data, and 8 - damped magnitude			
^3)^ GDAS: Global Data Assimilation System in 0.5° or 1° resolution			
^4)^ GFS: Global Forecast System in 0.25° resolution			

After obtaining trajectory results in the Step 1, we conducted further analysis using the trajectory results to obtain the frequency of passing trajectories over Chinese and Korean territories for backward and forward trajectories, respectively. For instance, to determine how many trajectories originated from Korea and reached Chinese territory within a week, a frequency analysis of the backward trajectory was performed through the following three steps. (Step 2–4 in Fig. 2):

In Step 2, using moth occurrence data collected from 18 different locations in Korea, rows from the HYSPLIT result data obtained in Step 1 were excluded for locations and times where the corresponding moth occurrence data indicated zero. This is to sort out the backward trajectories that are to be used for the next frequency analysis.

In Step 3, we used a blank raster layer with a spatial resolution of 0.1° to visualize the simulated trajectory frequency on a map. By overlaying the trajectory results on the blank raster layer, we counted the number of trajectories passing through each grid cell (*nhits*) and put it as an attribute variable of the final raster object.

In Step 4, the raster object made in the Step 3 was masked and cropped by the China administrative shape file obtained from the GADM (https://gadm.org/), and then the mean was calculated (*nhits_mean*) for each corresponding administrative district, or polygon object, of the shape file. By repeating the same process for every polygon, we obtained the *nhits_mean* values of all administrative districts, and then visualized them on the map.

We also conducted forward trajectory analysis in an attempt to compare the trajectory results and actual moth occurrence data in Korea. We used the same procedure as backward trajectory analysis, but this time, in the second step we used the data obtained from China to remove air trajectories where zero moths were captured. After obtaining the trimmed dataset, we visualized this on the Korea shape file according to the *nhits_mean* values of trajectories that pass over each administrative region.

#### Comparison of model simulation and moth occurrence data

We compared the model trajectory results from Step 1 and 2 and actual trap occurrence data, and the cases were divided into four categories (expressed as model result - trap data with O and/or X). In the model simulation, “O” indicates that the trajectory crossed the target area polygon, while “X” indicates that it did not. The trap data was also expressed as “O” or “X” meaning captured or not captured, respectively.

First, O-O cases indicate positive matching cases. In other words, O-O refers to instances where more than zero moths were captured in the trap, and the model trajectory traversed this location (*hit*) – like true positive cases. Secondly, in the case of O-X, the model simulates positive trajectory path over a location, indicating the plausible presence of moths in the trap, but no moth was actually captured (*no-hit*). We counted the number of O-O and O-X cases to calculate *hit ratio* (%). *Hit ratios* are summarized by the trap location and by the time period. The *hit ratio* by the trap location takes the number of O-O as numerator and the sum of O-O and O-X cases as denominator for each location. The *hit ratio* by the time period is the ratio of the O-O cases to the sum of O-O and O-X cases for each time period.

When it comes to X-O cases, it indicates the model predicts no trajectory path over the location, but more than zero moths were captured in the trap. Lastly, X-X cases indicate that the model predicted no trajectory path traversing the location, while zero moth was trapped in the location. These two cases could not be considered as *no-hit* or *hit*, respectively, because these cases likely occurred due to the lack of the spatial coverage of moth occurrence data both in China and Korea. Since we used the occurrence data only from 18 locations in Korea and five locations in China, the spatial coverage of ground-truth data was considerably limited. This results in inappropriate validation of the trajectory simulation results in the study. For example, the X-O cases in the backward trajectory analysis may become O-O if the moth occurrence data was available from other locations of Korea. Similarly, the X-X cases could not be considered as *hit*, since the backward trajectory from other unsampled locations of Korea could have passed over the trap location in China, resulting in O-X instead.

For the *hit* results (O-O) to be relayed to the subsequent phenotypic and genotypic validation of moth migration, further analysis was required to link individual trap locations, both in Korea and China, using backward and forward trajectory simulations for the same period. When backward trajectory simulation results and trap data match in one of the traps in China (O-O), we traced back the model simulation and identified one or more locations in Korea where the backward trajectory started. Subsequently, forward trajectory simulations using HYSPLIT were conducted from the *hit* location in China and the trajectory path was monitored whether it traversed the same locations in Korea where the backward trajectory simulation began. If there are matching locations for the same period, they were selected as candidate locations for an origin and a destination of migratory moths (marked as bold outline in Figs. 5 and 7).

## Results

### Data collection

**FIG. 3:**
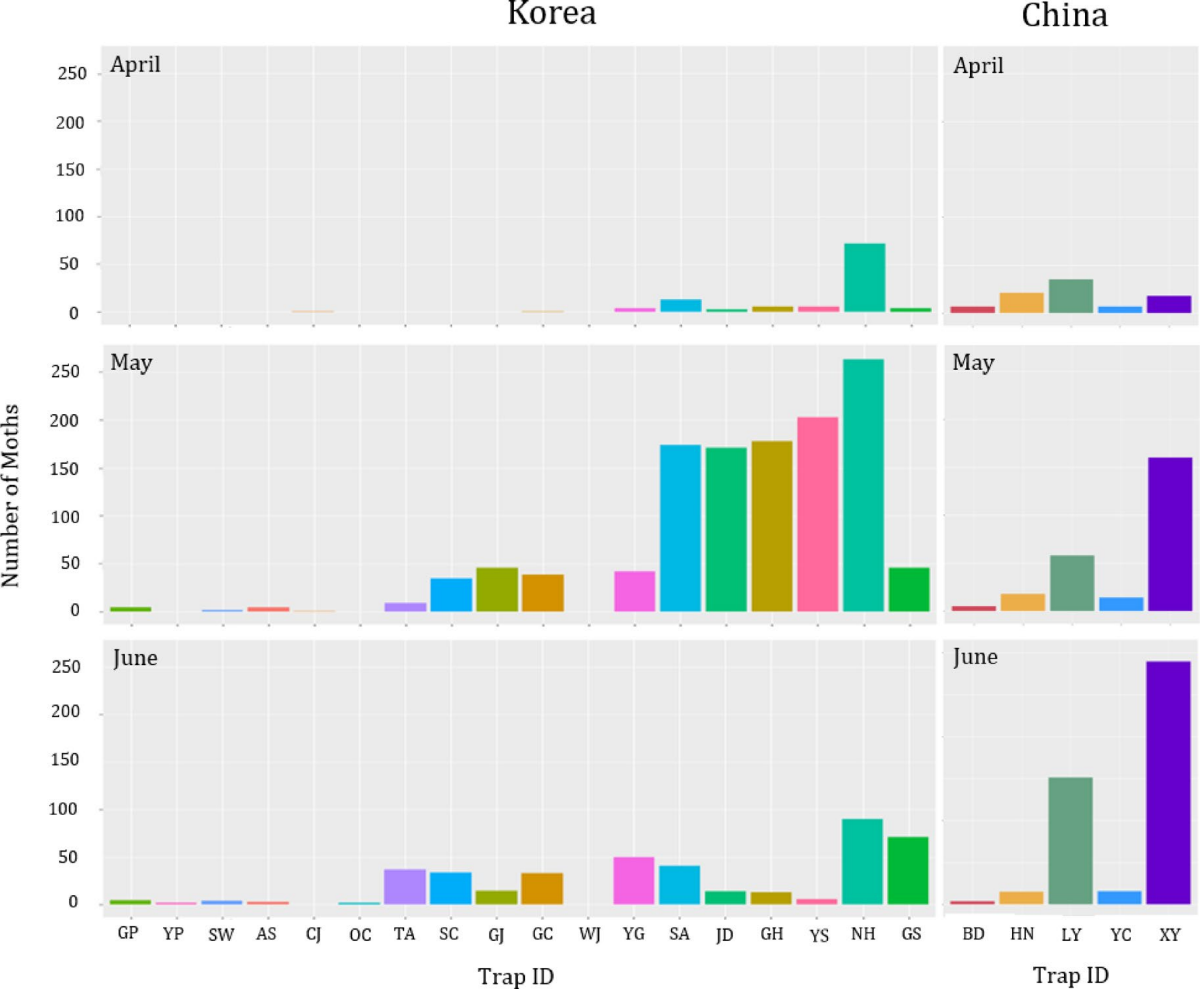
Moth occurrence data collected at 18 trap locations in Korea (three left panels) in Gimpo (GP), Yangpyeong (YP), Anseong (AS), and Suwon (SW) in Gyeonggi-do; Taean (TA) and Seocheon (SC) in Chungcheo ngnam-do; Cheongju (CJ) and Okcheon (OC) in Chungcheongbuk-do; Gimje (GJ), Wanju (WJ), and Gochang (GC) in Jeollabuk-do; Yeonggwang (YG), Shinan (SA), Jindo (JD), Goheung (GH), and Yeosu (YS) in Jeollanam-do; Namhae (NH) and Goseong (GS) in Gyeongsangnam-do, and at five trap locations in China (three right panels) in Luoyang Academy of Agricultural and Forestry Sciences (LY), Baoding Station (BD), Xinyang Railway Station (XY), Yancheng Railway Station (YC), and Modern agricultural science and technology experimental demonstration base of Henan Academy of Agricultural Sciences (HN) from April to June 2022. The x-axes are the trap ID, and y-axes are total number of moths captured each month in each location.

We collected *M. loreyi* occurrence data at 18 trap locations in Korea marked in Fig. 1. The occurrence data were analyzed to see the dynamics of the moth occurrences in April, May, and June, as shown in Fig. 3. Traps in NH have the largest total number of moths captured throughout the period. In April, there were only a few moths captured in the entire Gyeonggi-do, Chungcheongnam-do, Chungcheongbuk-do, and Jeollabuk-do, but Jeollanam-do and Gyeongsangnam-do (the southernmost part of Korea) had already started to witness the target moths. The most southern (SA, JD, GH, and YS) and western (NH) part of the Korean peninsula had the highest peaks of moth occurrence in May, but the numbers dropped again in June. SC in the southern part of Chungcheongnam-do, GJ and GC in Jeollabuk-do showed the same pattern but with smaller rate. The number slowly increased in TA, YG, and GS during the sampling period. Very few moths were captured in Gyeonggi-do (GP, YP, SW, and AS) and Chungcheongbuk-do (CJ and OC). WJ had zero number of moth occurrences during the data collection.

The three graphs on the right-hand side of Fig. 3 illustrate the spatial and temporal dynamics of moth occurrence in China during the same time periods (April, May, and June). BD showed persistently lowest number of *M. loreyi*, followed by HN and YC. LY showed the highest number of moths in April, then increased throughout the sampling period. Moth occurrences in XY (the southernmost trap location in China) also had an increasing pattern: it increased noticeably in May and June, having the highest peak in June.

### Backward trajectory from Korea

**Fig. 4:**
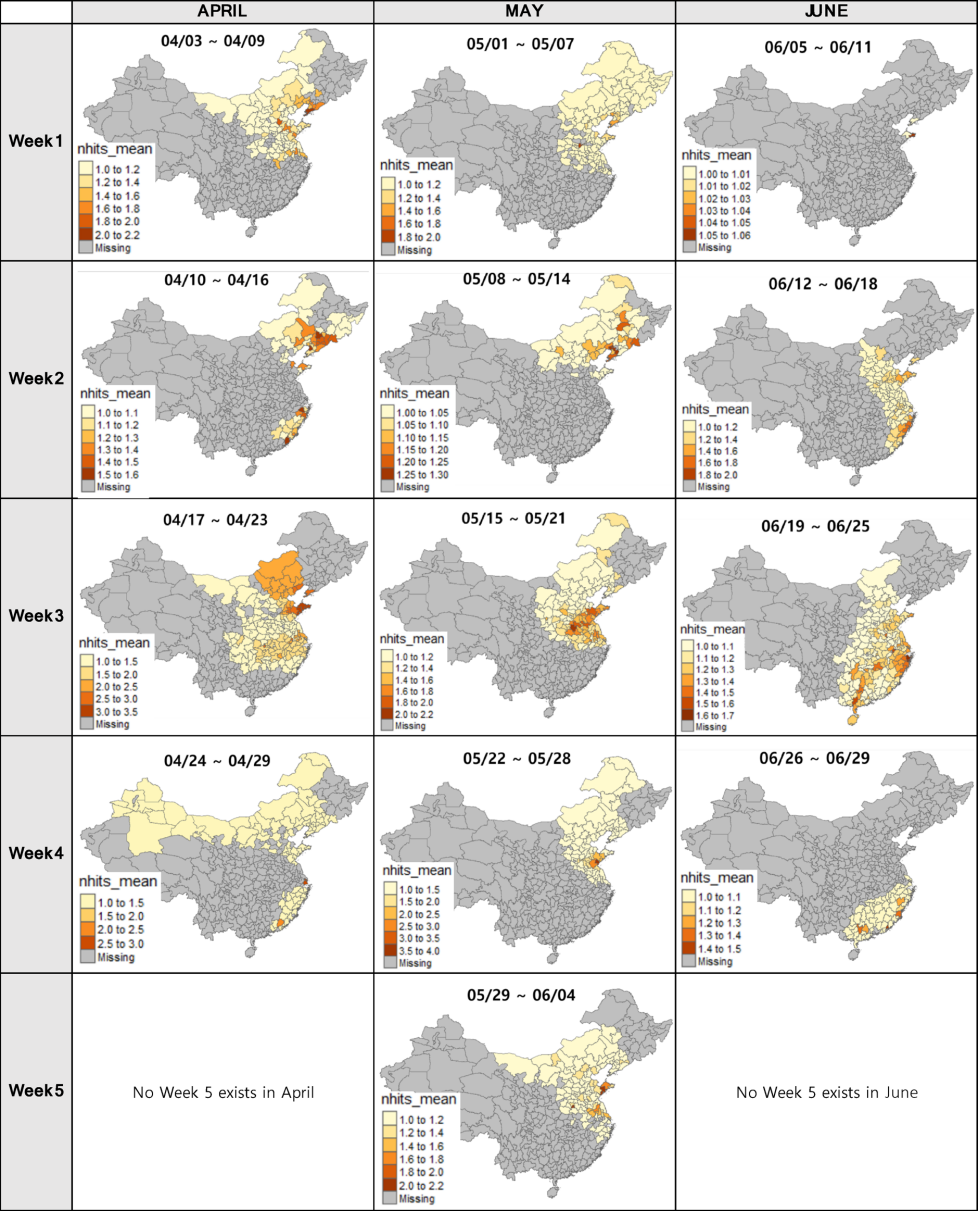
72-hour frequency analysis results over China from the backward trajectory (starting from 18 trap locations in Korea) model simulations from April to June. The color of the map indicates the average number of hits that go over the certain regions. The deeper red suggests the higher possibility to be the origin of moths. Grey color means that the regions are not likely the origin of moths, and each polygon is colored according to the *nhits_mean* values. The same color does not indicate the same value for different maps, because the ranges of value are different map by map.

Backward trajectory analysis from 18 trap locations in Korea was conducted to estimate the possible origins of *M. loreyi* in China. By running the HYSPLIT model between April and June for every 7 days, we acquired maps that visualize the average number of trajectory paths on the Chinese administrative boundaries with different colors indicating the frequencies of trajectory paths by the model (Fig. 4). The maps highlight the administrative areas with the highest *nhits_mean* value, which are referred to as *max_nhits*. This indicates that these areas have the greatest likelihood of being the origin of the moth during the specified period. The model simulations indicated that the moths captured in Korea during April, May, and June likely flew from the suggested areas: eastern, northeastern and southeastern China.

According to the backward trajectory model results from Korea (Fig. 4), there is a lack of prominent monthly patterns in the trajectory data, except for June when fewer areas exhibited positive trajectory patterns compared to April and May. In the first week of April, the trajectories encompassed the eastern and northeastern regions of China, displaying relatively even distribution. During the second week of April, the trajectories were divided into two parts of origin – in the northeastern China and southeastern part of China. During the third week, the paths of movement expanded in a southerly direction, encompassing both the central and eastern region. As the fourth week arrived, these paths reached into the northern parts of China as well as the southern edge. The simulation for May demonstrated similar patterns, with the trajectories primarily passing over the northeastern part of China during the first and second weeks. However, a shift in wind patterns led to a greater emphasis on the eastern part of China. In June, the trajectories predominantly covered the vertically elongated central and eastern parts of China during the second and third weeks. Finally, the last week of June showed the simulation concentration in the southern region of China.

### Comparison between the backward trajectory results and actual moth occurrence data in China

**Fig. 5:**
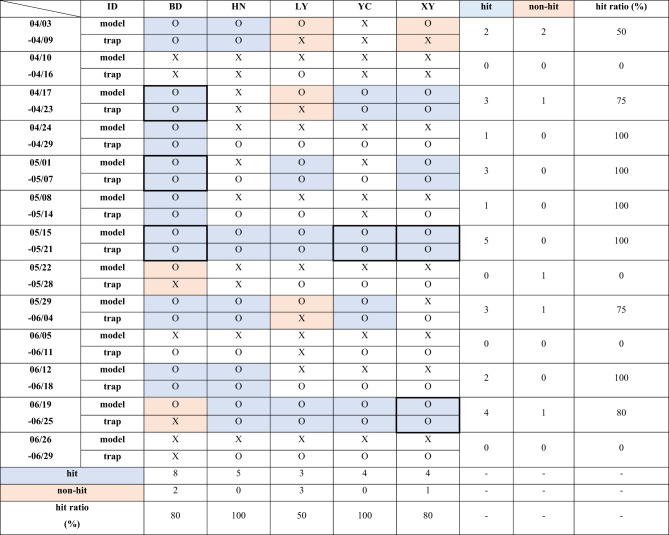
Comparison between backward trajectory model results arriving in China and moth occurrence data in China collected from each trap location in 2022. Blue shade indicates matches between the model results and dataset (O-O, *hit*), and red shade shows O-X where the model result was incorrect comparing with moth occurrence data (*no-hit*). Five columns are the trap locations in China (three right panels) in Luoyang Academy of Agricultural and Forestry Sciences (LY), Baoding Station (BD), Xinyang Railway Station (XY), Yancheng Railway Station (YC), and Modern agricultural science and technology experimental demonstration base of Henan Academy of Agricultural Sciences (HN) from April to June 2022. The cells in bold lines are the candidate locations of moth origin that match with destinations in Korea, resulting from backward and forward trajectory analyses.

We made comparisons of the backward trajectory results of the HYSPLIT (Fig. 4) with the daily trap occurrence data collected in China (Supplementary Fig. 1) in 2022, and calculated the *hit ratio* by the location and time period, respectively (Fig. 5). Using the method shown in Data and methods section, we only extracted the O-O cases (*hit*) and O-X cases (*no-hit*) for the *hit ratio* calculation, but discarded the cases of X-O and X-X for this analysis. As a result, LY showed a *hit ratio* of about 50%, BD and XY traps had a *hit ratio* of around 80% by location, while HN and YC showed 100% *hit ratio*. This result indicates when trajectories from 18 trap locations in Korea passed the five trap locations in China, the *M. loreyi* was actually captured in those five traps in most cases. It is important to note that the maps in Fig. 4 include all the possible trajectory pathways in China. The moths traveled from south to north within China.

The *hit ratios* by the time period were 100% during the period of 05/15–05/21 in all five locations in China (Fig. 5), indicating that comprehensive occurrence of *M. loreyi* took place and thus these periods need to be focused for the subsequent migration validation in this time period. The periods of 04/24 − 04/29, 05/01–05/07, and 05/08–05/14 also exhibit 100% *hit ratio* with total number of O-O and O-X combined lower than 5 for each period. The periods of 04/03–04/09, 04/17–04/23, 05/29 − 06/04, 06/12 − 06/18, and 06/19 − 06/25 showed *hit ratios* that equals to or are greater than 50% in all or part of locations, while period of 04/10–04/16, 05/22–05/28, 06/05–06/11, and 06/26–06/29 had 0% of *hit ratio*, indicating that it was impossible to calculate the *hit ratios* due to the absence of O-O and/or O-X cases.

### Forward trajectory from China

**Fig. 6:**
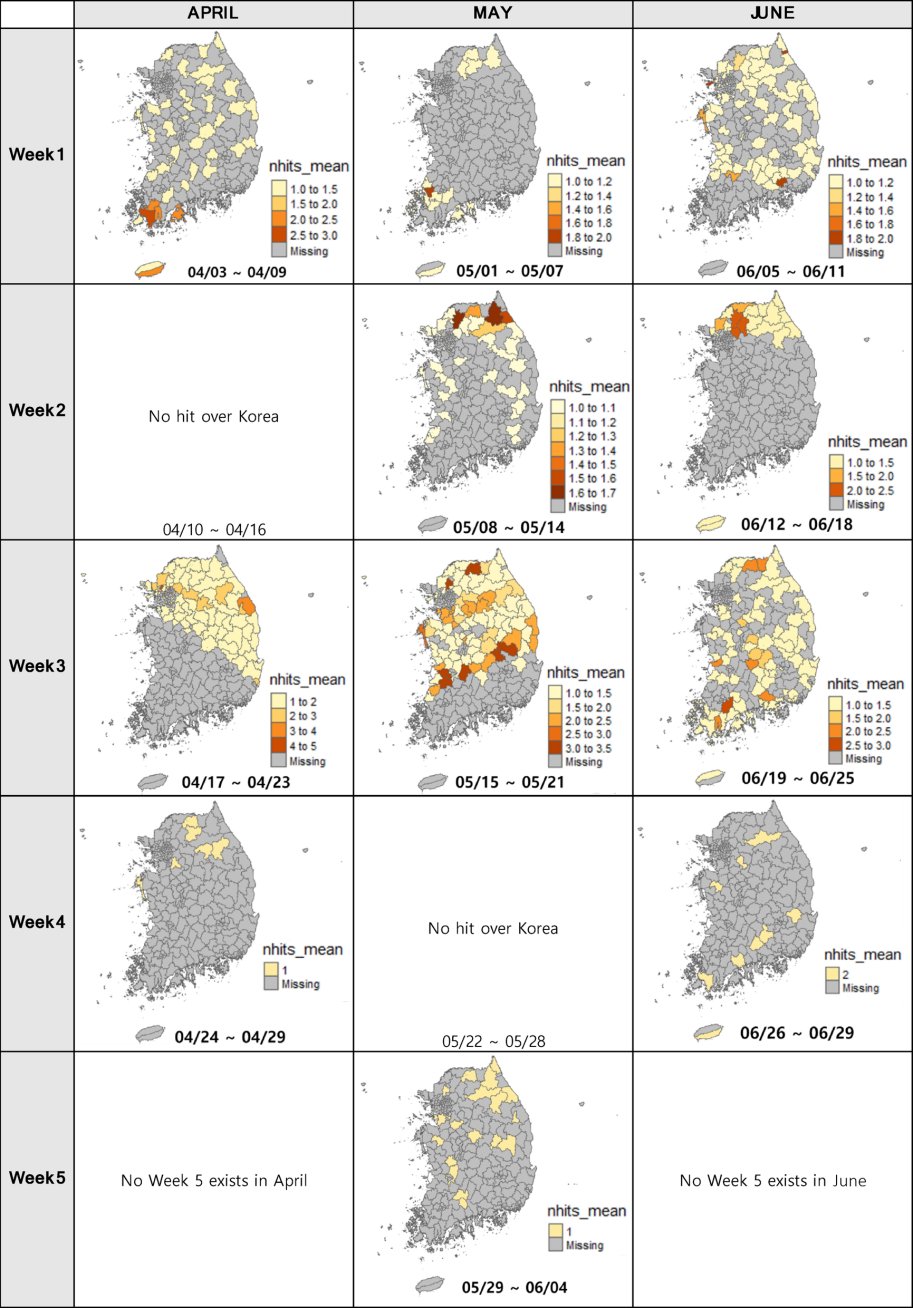
72-hour frequency analysis results over Korea from the forward trajectory (starting from five trap locations in China) model simulations for *M. loreyi* from April to June. Grey color means zero *nhits* in the location and other colors indicate the certain number of *nhits* according to the figure legend. The colors represent the average number of trajectories that passed through the individual administrative areas.

Forward trajectory analysis was also conducted by utilizing the moth occurrence data from five locations in China and run the HYSPLIT model to get the potential trajectory paths over Korea. Applying the same methods as the backward trajectory analysis, we created forward trajectory results for *M. loreyi* from five trap locations in China, as shown in Fig. 6. As a result, we acquired multiple maps that visualize the number of trajectory paths on the Korean administrative boundaries with different colors indicating the frequencies of trajectory paths by the model over Korean territory.

Compared to the backward trajectory results over China (Fig. 4), the forward trajectory maps (Fig. 6) showed more discrete patterns, which clearly suggests the scarcity of the moth occurrence data from China. During the first week of April, trajectories exhibited an irregular pattern throughout the country, with particular emphasis on Southwestern edge of Korea, especially Haenam-gun, Jeollanam-do, where the NH trap is installed. In the third week of April, trajectories appeared to have traversed the northern and eastern regions (mostly in Gyeonggi-do, Gangwon-do, and Gyeongsangbuk-do) of Korea, while in the fourth week, the occurrence of trajectories passing was infrequent - only six districts (including Taean where TA trap is located) have *nhits_mean* that is higher than zero. In the first week of May, there was an evidence of trajectory occurrences in regions across both the northern (Gyeonggi-do and Gangwon-do) and southern (Jeollanam-do) parts of Korea, with a notable concentration of trajectories and higher *nhits_mean* values observed in the south. During the second week of May, a relatively higher number of trajectories were observed passing through the northern regions (Gyeonggi-do and Gangwon-do) and some districts in Chungcheongnam-do, Jeollanam-do, and Jeollabuk-do, while in the third week, the trajectories are extensively distributed across the country except for Jeollanam-do and Gyeongsangnam-do. In the last week of May, trajectories were scattered throughout the country, except for the southeastern part. Trajectories exhibited a widespread distribution across the entire country in the first week of June, with the exception of Jeollanam-do. In the second week, trajectories passed through the northern part of Gyeonggi-do and Gangwon-do, and also reached Jeju-do. As for the third week of June, trajectories traversed the entire country, displaying a broad coverage across different regions. However, by the last week of June, trajectory paths were limited to specific regions, indicating a more localized distribution. In the second week of April and the fourth week of May, forward trajectories originating from China did not cross Korean territory, likely due to the absence of initial starting points; consequently, they are not represented in the figure.

### Comparison between the backward trajectory results and actual moth occurrence data in China

**Fig. 7:**
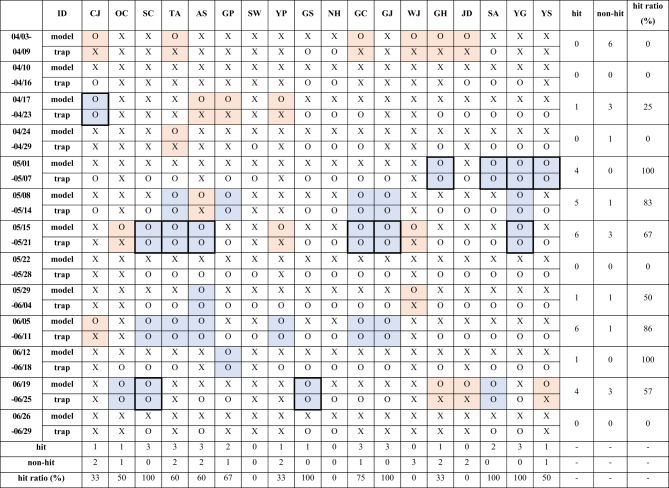
Comparison between forward trajectory results arriving in Korea and moth occurrence data in Korea collected from each trap location in 2022. Blue shade indicates matches between the model results and dataset (O-O, *hit*), and red shade shows O-X where the model result was incorrect comparing with moth occurrence data. The column names indicate the trap locations in Gimpo (GP), Yangpyeong (YP), Anseong (AS), and Suwon (SW) in Gyeonggi-do; Taean (TA) and Seocheon (SC) in Chungcheongnam-do; Cheongju (CJ) and Okcheon (OC) in Chungcheongbuk-do; Gimje (GJ), Wanju (WJ), and Gochang (GC) in Jeollabuk-do; Yeonggwang (YG), Shinan (SA), Jindo (JD), Goheung (GH), and Yeosu (YS) in Jeollanam-do; Namhae (NH) and Goseong (GS) in Gyeongsangnam-do. The cells in bold lines are the candidate locations of moths’ migratory destinations that match with origins in China, resulting from backward and forward trajectory analyses.

Forward trajectory model simulation results starting from the five locations in China were compared with the actual moth occurrence data at the 18 sites in Korea (Fig. 7). By conducting the comparison, SC, GS, GJ, SA, and YG traps showed the highest *hit ratio* (100%), where the simulated trajectory paths corresponded to the actual moth occurrence data (O-O). Meanwhile, NH had 0% of *hit ratio* where all of the model simulation and moth occurrence data results were X-O. In other words, all the moths captured in NH might be from locations other than the five data collection sites used in this study. SW, WJ, and JD also showed 0% *hit ratio*, but this time they are mixed with O-X, X-O, and X-X. Especially, the O-X cases in the three traps mean that the model erroneously simulated moth presence in this region, despite no moth being captured in reality. The *hit ratio* by time is 100% during 05/01–05/07 and 06/12–06/18, and 0% for 04/03–04/09, 04/10–04/16, 04/24–04/29, 05/22 − 05/28, and 06/26 − 06/29.

In summary, we compared the model simulation results and actual trap data for China (Fig. 5) and Korea (Fig. 7), and identified the candidate locations for an origin and a destination by matching trap locations and time periods in both countries. As a result, we narrowed down trap locations where *hit* occurred in both countries during corresponding time periods (bold lined cells in Figs. 5 and 7). During the period of 04/17–05/21, it was indicated that the moths captured in CJ, Korea, might be related to the ones found in the trap of BD in China. In the same way, it was recommended that researchers compare the phenotypic and genotypic characteristics of the moths captured in GH, SA, YG, and YS, Korea with the ones in BD, China, for the period of 05/01–05/07. In addition, multiple locations, marked with bold lined cells in Figs. 5 and 7 for the periods of 05/15–05/21 and 06/19–06/25, were identified as the candidate locations for further validation as well.

## Discussion

The primary objective of this research was to find the feasibility of utilizing HYSPLIT trajectory analyses in identifying the potential origins of moths, thus to streamline the subsequent model validation process. Implementing this process before validation may result in a considerable reduction in the required efforts and resources for validation. The model simulated potential trajectories of migratory moth species and was evaluated by comparing the model results with actual moth occurrence data at the destination points of the trajectories. The *hit* and *non-hit* classifications (O-O and O-X, respectively) in Figs. 5 and 7 were utilized to determine the model’s *hit-ratio* based on both trajectory path and time period. If the comparison between model results and actual data at a trap location resulted in *hit* (O-O case) in one country, the result was subsequently linked to *hit* locations in another country using backward and forward trajectory simulations for the same period. In the study, we successfully identified multiple sets of most likely origin and destination locations and time periods as target moth samples for the following phenotypic and genotypic validation.

There are some expected benefits when using HYSPLIT model for migratory species analysis. Predicted trajectories will be capable of increasing the efficiency of the validation process by choosing the most likely origin according to the model results. Thus, governments and research institutes could make use of this model to minimize potential losses by focusing their efforts on targeted areas during moth sampling campaigns based on the model’s results. This targeted validation through the HYSPLIT trajectory modelling will not only help prioritize the locations to obtain moth samples, but also reduce the time and resources for the subsequent validation. In addition, accurate validation of trajectory models allows for more effective pest control strategies by predicting impact regions, resulting in better crop protection while minimizing environmental impact. Moreover, when the insect parameters added to the online HYSPLIT model are also released as an R script, further research should add it to the present model to add more precision to the model simulation results. With improved model simulations, the results become more reliable, which could benefit the economy by optimizing resources required to collect moth samples that are needed for validation. It can also be ecofriendly by minimizing pesticide use.

The HYSPLIT model could falsely simulate trajectory. One of the problems when using the HYSPLIT model is that it does not include insect parameters in the model, which could devalue the model simulation results and falsely simulate the trajectory – the moths do not follow the simulated trajectory. To address this issue, it is recommended to use other supporting tools, such as the integrated entomological radar-trajectory analysis model. As some papers already mentioned^13^, integrating radar data potentially enhanced model simulation results by providing entomological data. This data, for example moth flight direction, speed, and altitude, can help identify better moth migration pattern analyses by applying moth activities instead of assuming passive flight behaviors of moths. Moreover, climate change alternate flight patterns by poorly resolving monsoon which is mesoscale. The reliance of HYSPLIT on GDAS data could falsely represent monsoon patterns due to the low resolution (0.5° or 1°)^18^. This can neglect the boundary layer turbulence and alternate local monsoon wind systems during monsoon and monsoon transition seasons.

Trajectory model validation is challenging, although it is essential to ensure the model’s reliability. Therefore, the species identification should be integrated to encompass genotype as well as phenotype analyses. They are able to offer evidence related to the moths’ origins, such as molecular markers, radar, and mark-recapture^19^. It is primary to check if the target moths were captured in the trap at the destination location to validate the model effectively, but at the same the target moth species has to be found in the trap at the starting point as well. Therefore, a subsequent analysis to determine the genetic diversity within the two groups of moths (in the starting point and the destination) should be conducted to see if they are genetically similar. Through the above-mentioned process of validation, researchers can validate the model prediction results in a comprehensive way.

There are several validation methods that have been employed to ensure the accuracy and reliability of the trajectory models. 13 Spodoptera species were obtained from Genebank to compare them with Fall armyworm, *Spodoptera frugiperda* species in Ecuador by using genotyping method using mitochondrial cytochrome oxidase subunit I (COI) and nuclear triose phosphate isomerase genes (Tpi)^20^. Phenotyping (morphological characteristics in the wing pattern and male genitalia) as well as genotyping (partial mitochondrial DNA sequences of cytochrome c oxidase subunit 1 gene) methods were used to identify different species^5^. Genetic variation of fall armyworm populations in Africa (DR Congo, Tanzania, Uganda, and Zimbabwe) and Asia (Bangladesh, Korea, Nepal, and Vietnam) were analysed using two genes - Tpi and COI^21^. In this study, they found similar genetic diversity between the two regions, indicating that the spread of fall armyworm from Africa to Asia did not involve significant genetic changes. The Loop-Mediated Isothermal Amplification (LAMP) assay was also utilized as a faster, simpler, and more effective identification method^4^. There is a review study that utilized pollen and the pollination ecology of the moth species to track behaviors of moths^22^. These kinds of research require a lot of resources as well as active interactions between countries, which become big hurdles. Our model can help determine a plausible origin of the moths that could be compared and analyzed with moth samples in a destination.

In this study, we compared the model simulation results and actual trap data in China (Fig. 5) and Korea (Fig. 7). Using this in-depth analysis comparing individual locations and time periods in two countries, we narrowed down candidate locations for further phenotypic and genotypic validation of moth migration (bold lined cells in Figs. 5 and 7). For instance, as the moths captured in SC of Korea during the period of 05/15–05/21 were matched with the ones captured in XY, China, they became candidate locations for further validation analysis. As an exemplary case, our joint research team conducted haplotype-based genotypic analysis between the SC moth samples and all haplotypes of *M. Loreyi* reported in China. It was found, as a result, that a haplotype of moth captured in SC, Korea, has the same genotype with one of haplotypes from China (personal commun.), possibly indicating that they migrated to Korea with air currents. Although further systematic analysis is required to collect stronger evidence of the moth migration in between China and Korea, this finding supports the objective of our study that it is feasible to use the HYSPLIT-based trajectory analysis and the moth trap data to support the targeted validation of moth migration by narrowing down some locations for subsequent phenotypic and genotypic characterization of the trapped moths in both locations.

Although the benefits follow when using this model and the validation work, researchers might face drawbacks from low spatial resolution of moth occurrence data. Data collection from every single region/citie of the entire country is impossible due to the limited time and resources. The habitat of *M. Loreyi* is distributed extensively, mainly in the southern and eastern part of China, thus using only five data collection sites for model validation is clearly insufficient, which can also be observed from the discrete pattern in Fig. 5. Enhancing spatial resolution requires an active collaboration with the Chinese government to acquire national/multi-regional data^23,24^. It is paradoxical that this model tries to improve the cost-effectiveness when conducting model validation and streamlining the sampling area, but at the same time it necessitates ample initial input data to increase model reliability.

Furthermore, it is important to note that the mean winter temperatures in most regions of China are so low that the moths can only survive and overwinter in the southernmost parts of the country^25,26^. This implies that the moths probably migrated with jet stream in the upper level of the atmosphere from the southern China to the regions where the model simulated (Fig. 4). Previous studies provide clear clues showing there are seasonal migration of certain insect species within the Chinese territory. Although studies about *M. loryei* is lacking, other studies showed that the species similar to *M. loreyi*, such as *Mythimna separata*, migrate from south to north during spring (March to mid-April)^27^. Genetic structure and demographic history of Diamondback moth in China was studied, and it is revealed that they primarily migrate from South to North in spring and summer^25^. Another trajectory simulation research on invasive fall armyworm in eastern manifested that this kind migrates from Southeastern China or other countries in Indochina, such as Thailand, Vietnam, and Laos, to Huang-Huai-Hai region (mainly Henan, Shandong and Hebei) and Northeastern China throughout spring and summer^28^. It was also shown that the Korean Peninsula is also a suitable location for summer-breeding populations.

Our study holds the potential to enhance the cost-effectiveness of migratory moth trajectory research. Addressing the inherent limitations would be advantageous, as this model would make future studies on moth migratory trajectories more feasible and cost-effective. Also, international and nation-wide research collaboration could have an indirect effect on job creation on plant protection in response to invasive species^23^. A possible future work needs to include not only overcoming challenges of this study, but also facilitating migratory pest moth species early warning systems by using weather forecast data instead of observation data. The anticipated contribution of this study lies in its potential to support the Integrated Pest Management (IPM) system by mitigating the potential harm caused by invasive migratory pest species to major crops.

## Electronic supplementary material

Below is the link to the electronic supplementary material.

Supplementary Material 1

## Data Availability

All the weather data used in this study can be accessed from the internet sources mentioned in the text. The moth trap data can be obtained by request to the corresponding author.
