# Supplementary material for: Trajectory analysis of Mythimna Loreyi migration into Korea using the HYSPLIT model
Source: Sci Rep. 2025 Jul 1;15:21486. doi: 10.1038/s41598-025-07876-9 (PMC12215364; doi:10.1038/s41598-025-07876-9)
Supplement: Supplementary file 1 — Supplementary Material 1 [file 41598_2025_7876_MOESM1_ESM.pdf]

Supplementary Information for

Trajectory analysis of *Mythimna loreyi* migration into Korea using the HYSPLIT model

Juhyeong Han[1], Sunghoon Baek[2], Xue-Jing Wang[3], Chun-Sen Ma[3], Kwang-Hyung Kim[1,4,5]*

[1]Interdisciplinary Program in Agricultural and Forest Meteorology, Seoul National University, Seoul 08826, Korea;

[2]Department of Industrial Entomology, Korea National College of Agriculture and Fisheries, Jeonju, Korea;

[3]School of Life Science, Institutes of Life Science and Green Development, Hebei University, Baoding 071002, People's Republic of China

[4]Department of Agricultural Biotechnology, Seoul National University, Seoul 08826, Korea

[5]Research Institute of Agriculture and Life Sciences, Seoul National University, Seoul 08826, Korea

*Corresponding Author: Kwang-Hyung Kim, Department of Agricultural Biotechnology, Seoul National University, Room 5122, Seoul 08826, Korea, E-mail: sospicy77@snu.ac.kr

Supplementary Figures

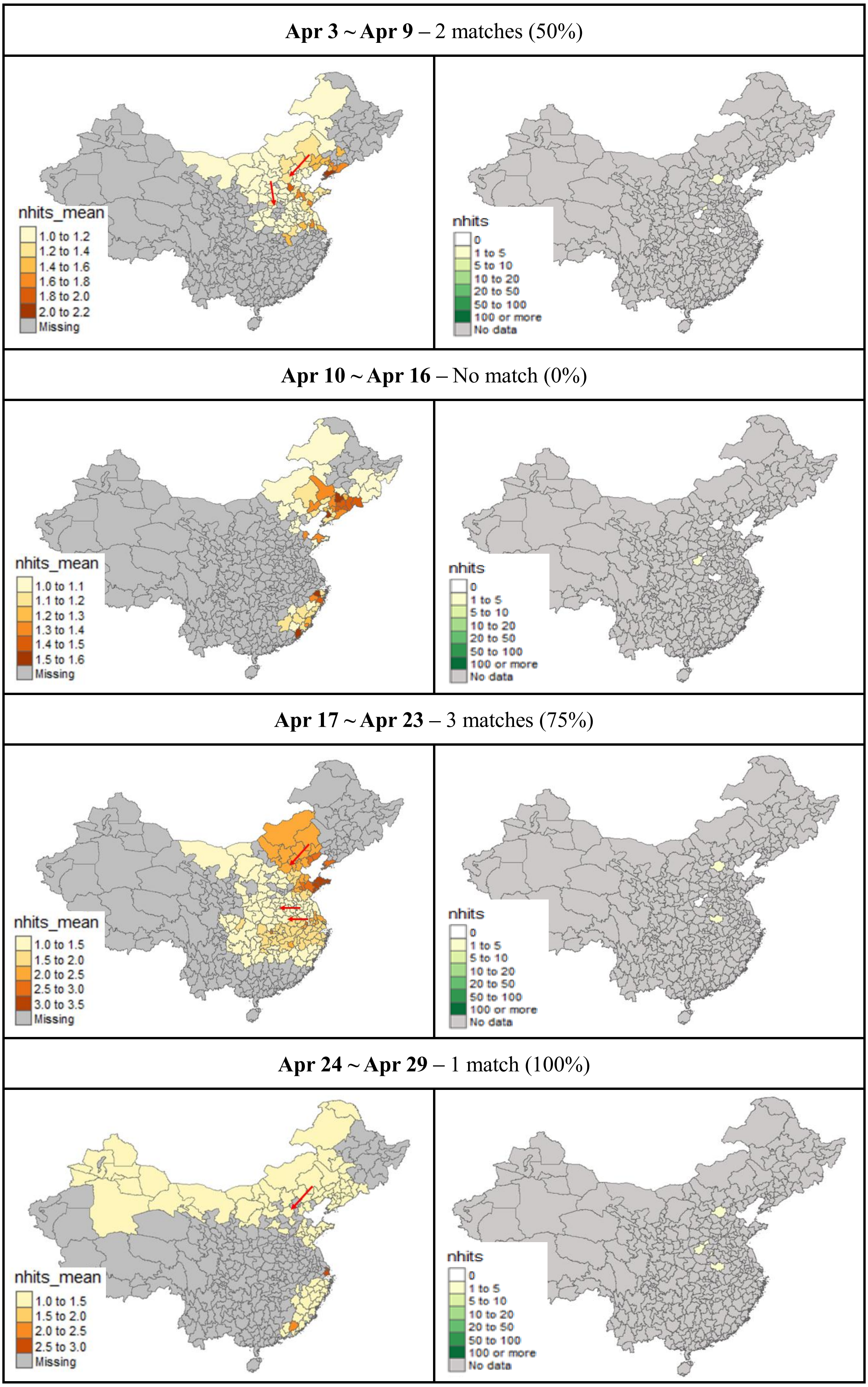

May 1 ~ May 7 – 3 matches (100%)

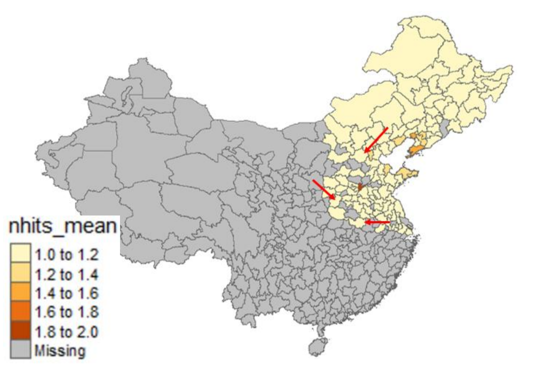

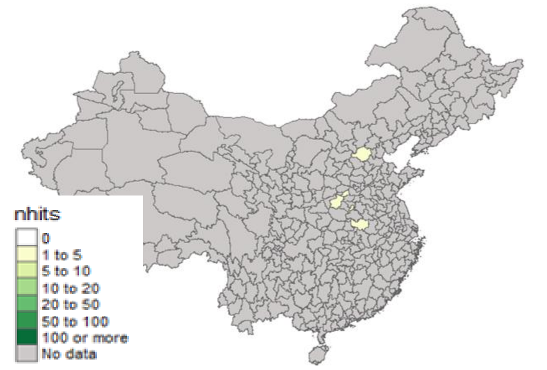

May 8 ~ May 14 – 1 match (100%)

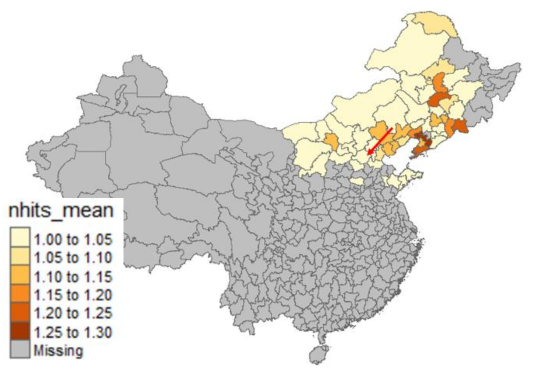

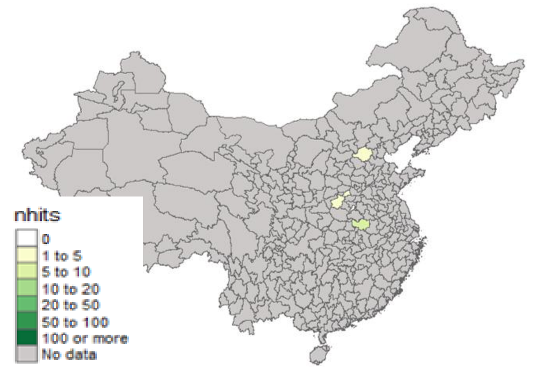

May 15 ~ May 21 – 5 matches (100%)

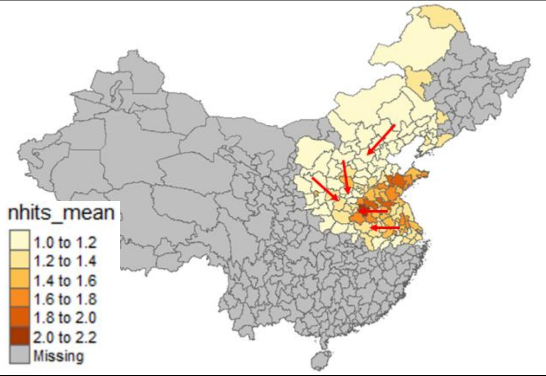

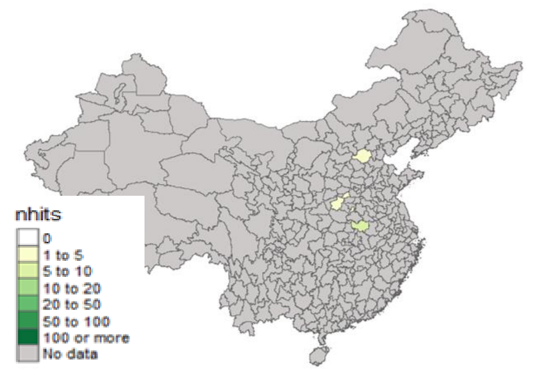

May 22 ~ May 28 - No match (0%)

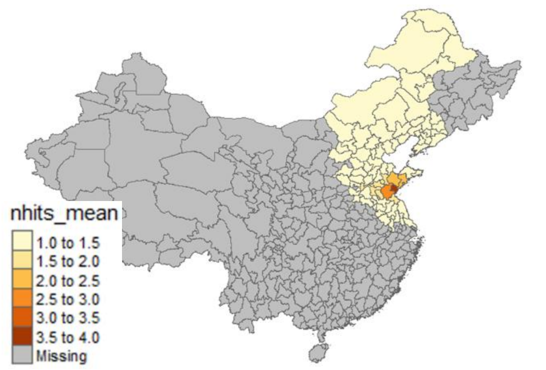

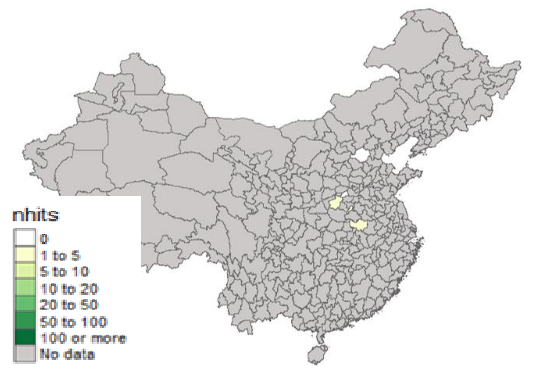

May 29 ~ Jun 4 – 3 matches (75%)

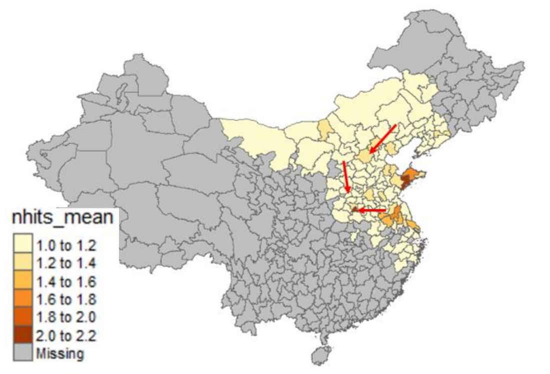

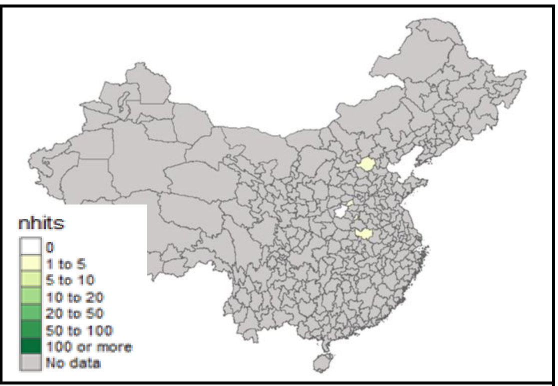

Jun 5 ~ Jun 11 - No match (0%)

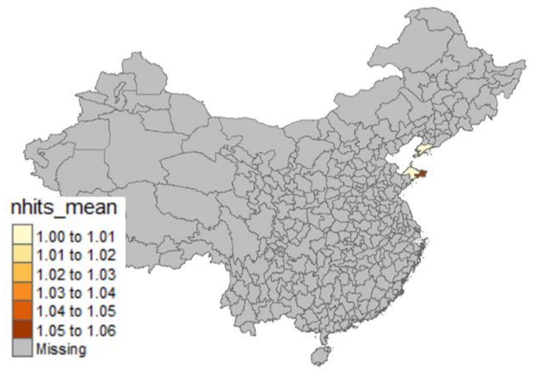

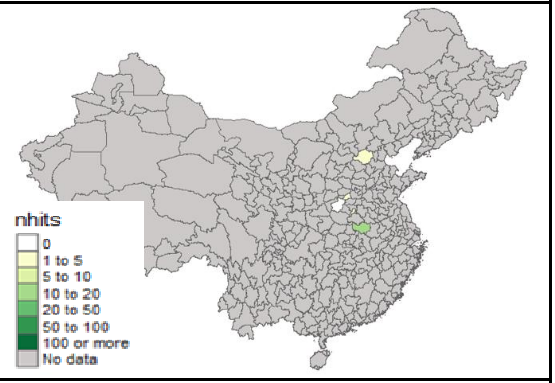

Jun 12 ~ Jun 18 – 2 matches (100%)

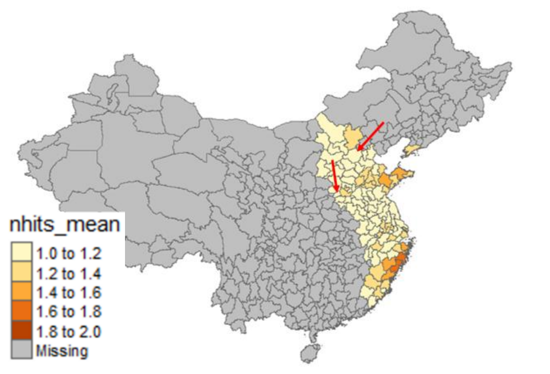

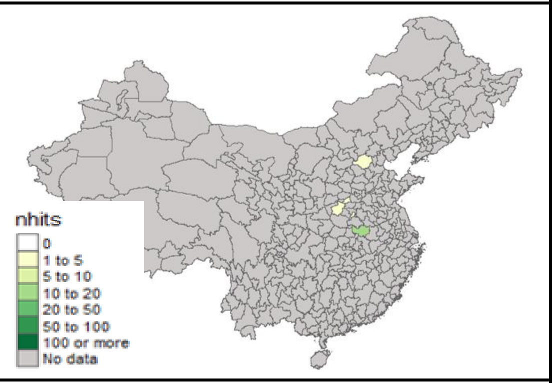

Jun 19 ~ Jun 25 - 4 matches (80%)

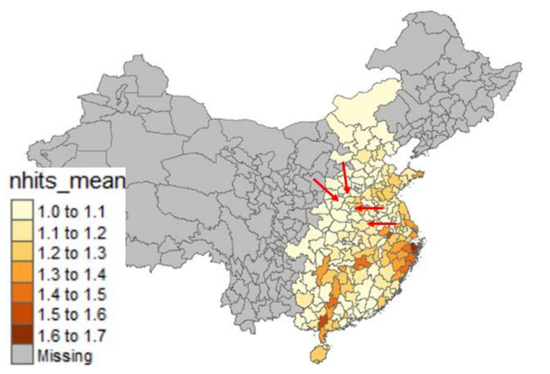

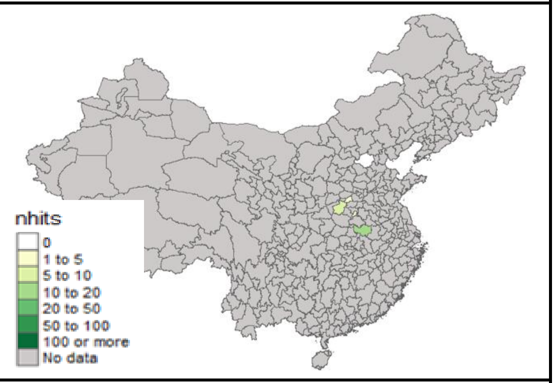

Jun 26 ~ Jun 29 – No match (0%)

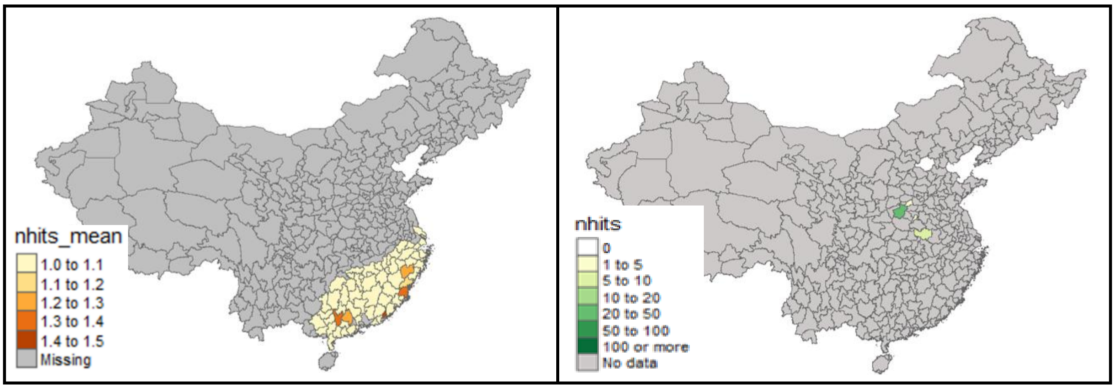

Supplementary figure 1. Comparison between HYSPLIT model backward simulation (left) and moth capture data (right) for each week. Red arrows on the figure on the left indicate the trap locations where the model simulation and actual data match. The match is when the model accurately replicated the situation, i.e. when the target moth was captured in a trap sites, the modeled trajectories also intersected with the trap area. In the image on the left-hand side, polygons are shaded anything but grey if they were crossed by at least one trajectory, while grey indicates the absence of any trajectory crossings. In the image on the right-hand side, polygons are grey in areas where no sampling took place, white if no moths were captured during that period, and other colors if at least one moth was captured.
